# Modified bolster dressing with continuous suction improves skin graft survival for an oral cavity wound

**DOI:** 10.1186/s40463-018-0314-7

**Published:** 2018-11-14

**Authors:** Eunkyu Lee, Song I Park, Donghyeok Kim, Hokyung Jin, Han-Sin Jeong

**Affiliations:** Department of Otorhinolaryngology-Head and Neck Surgery, Samsung Medical Center, Sungkyunkwan University School of Medicine, 81 Irwon-ro, Gangnam-gu, Seoul, 06351 Republic of Korea

**Keywords:** Skin graft, Mouth defects, Saliva, Suction drain

## Abstract

**Background:**

Skin engraftment of intraoral defects is known to be inconsistent due to mobility of the oral structure, uneven wounds, and accumulation of saliva under the skin graft. To improve the success rate of oral skin graft, we proposed a novel and simple dressing technique for intraoral skin graft to control saliva accumulation, in comparison with the conventional bolster dressing.

**Methods:**

We retrospectively reviewed 31 patients reconstructed with skin graft in their intraoral defect. The patients were divided into two groups; conventional bolster group (*n* = 21) and modified bolster group (*n* = 10). In the modified bolster group, a polyvinyl alcohol sponge was designed to fit the skin graft and a suction drain was inserted with tagging suture to apply continuous suction. We analyzed the success rate, the size of the skin grafts and clinical variables of each method.

**Results:**

The overall success rate of oral skin graft was not different between the two groups (90.0 and 90.5%). However, partial necrosis in the engrafted skin was observed frequently in the control group (57.1% versus 20.0%). The relative engrafted area was significantly higher in the modified bolster group (55.0 ± 11.6% versus 23.0 ± 18.7%, *p* = 0.015). The duration of bolster dressing and the time to start an oral diet were shorter in the modified bolster group.

**Conclusions:**

Our modified method could be easily applied for removing saliva accumulation under a skin graft and for enhancing skin engraftment of an oral cavity wound.

**Electronic supplementary material:**

The online version of this article (10.1186/s40463-018-0314-7) contains supplementary material, which is available to authorized users.

## Background

Intraoral defects can be reconstructed with various methods depending on different factors, including subsite and extent of defect, patient factors, surgical skills and institutional resources. Skin graft is a useful method for repairing wound, particularly for superficial defects that could not be closed primarily. It is a simple and less time-consuming procedure compared to regional and free flaps. Transplanted skin graft can protect the wound bed from further trauma and provide an important barrier to infection [1]. To achieve successful survival of skin graft for wound healing, regenerating and restoring blood supply into the grafted skin is critical during the immediate postoperative period by immobilization of skin graft and removal of contaminants [1–5].

Different from other organs, an intraoral wound has some disadvantages in skin engraftment due to constant mobility of the oral structure, uneven wound bed, and accumulation of saliva between grafted skin and wound bed [6]. To overcome these disadvantages, various methods have been tried to secure intraoral skin graft for immobilization [7–9]. Many types of stents, such as simple cotton ball, resin molds, and foam pad have been used in a tie-over bolster technique to anchor the graft to the wound bed [10].

However, few trials have been conducted to control undesirable accumulation of saliva under intraoral skin grafts. Saliva aids intraoral wound healing by providing a humid environment and it contains many growth factors [11]. However, accumulation of saliva in a potential dead space under the skin graft could result in graft-bed separation, eventually leading to graft failure. Therefore, adequate control of saliva is one of the important factors to improve the survival of intraoral skin graft. In this article, we proposed a novel and simple technique for intraoral skin graft to control saliva effectively with a conventional bolster dressing.

## Methods

### Study patient

We retrospectively reviewed prospectively enrolled head and neck cancer patients in our institution. All patients submitted written informed consent for use of their clinical and biological data. The study protocol was approved by our Institutional Review Board (Protocol No. 2010-05-090, 2015-06-132, ClinicalTrials.gov NCT02546895). From 2014 to 2017, a total of 31 patients who had undergone skin graft reconstruction for oral cavity defect were reviewed. Of 31 patients, 21 used conventional bolster dressing (without continuous suction) and 10 used our modified bolster dressing. Clinical data including patient age, gender, pathology, primary tumor site, clinical and pathological stages, and the sizes of defect and graft were collected. Gross photographs of the intraoral wound and the medical record were used to analyze successful engraftment of the skin graft. We analyzed the success rate and size of skin graft, duration of bolster dressing, and the time start of an oral diet from operation day.

### Surgical indications for skin graft reconstruction

We selected patients with newly diagnosed oral cavity cancer under cT1–2 as candidates for skin graft reconstruction. The use of skin graft was decided by a responsible surgeon on the basis of the extent of defect in the oral cavity. If the size and depth of the tumor seemed to be more than 6 or 7 cm and 1 cm respectively, we preferred flap reconstruction rather than skin graft. In one case (No. 7 in the conventional bolster group, Additional file 1: Table S1), the tumor involved the tongue and the floor of the mouth (the clinical stage of cT3). However, primary closure was done for the tongue lesion so that we could apply skin graft to the remaining defect of the floor of the mouth.

Skin grafts were harvested with both full thickness and split thickness methods. It is known that the full thickness method has better esthetic results and less contracture than the split thickness method [12]. In our study, though split thickness (12/1000 in.) skin graft tended to be used for relatively large defects, there was no strict criteria for the choice of skin graft.

### A conventional bolster method for skin graft

After resecting the primary tumor, a full thickness or split thickness skin graft was harvested from the patient’s inguinal or thigh area and placed on a defect of the floor of mouth or tongue (surgically resected area) with interrupted sutures at the graft edge. The surface of the skin graft was processed with the pie-crust technique. A polyvinyl alcohol sponge (Merocel®, Merocel Co, CT, USA) was designed to fit to skin graft size and applied directly with tie-over sutures to compress the skin graft.

### A modified bolster technique of skin graft

**Fig. 1:**
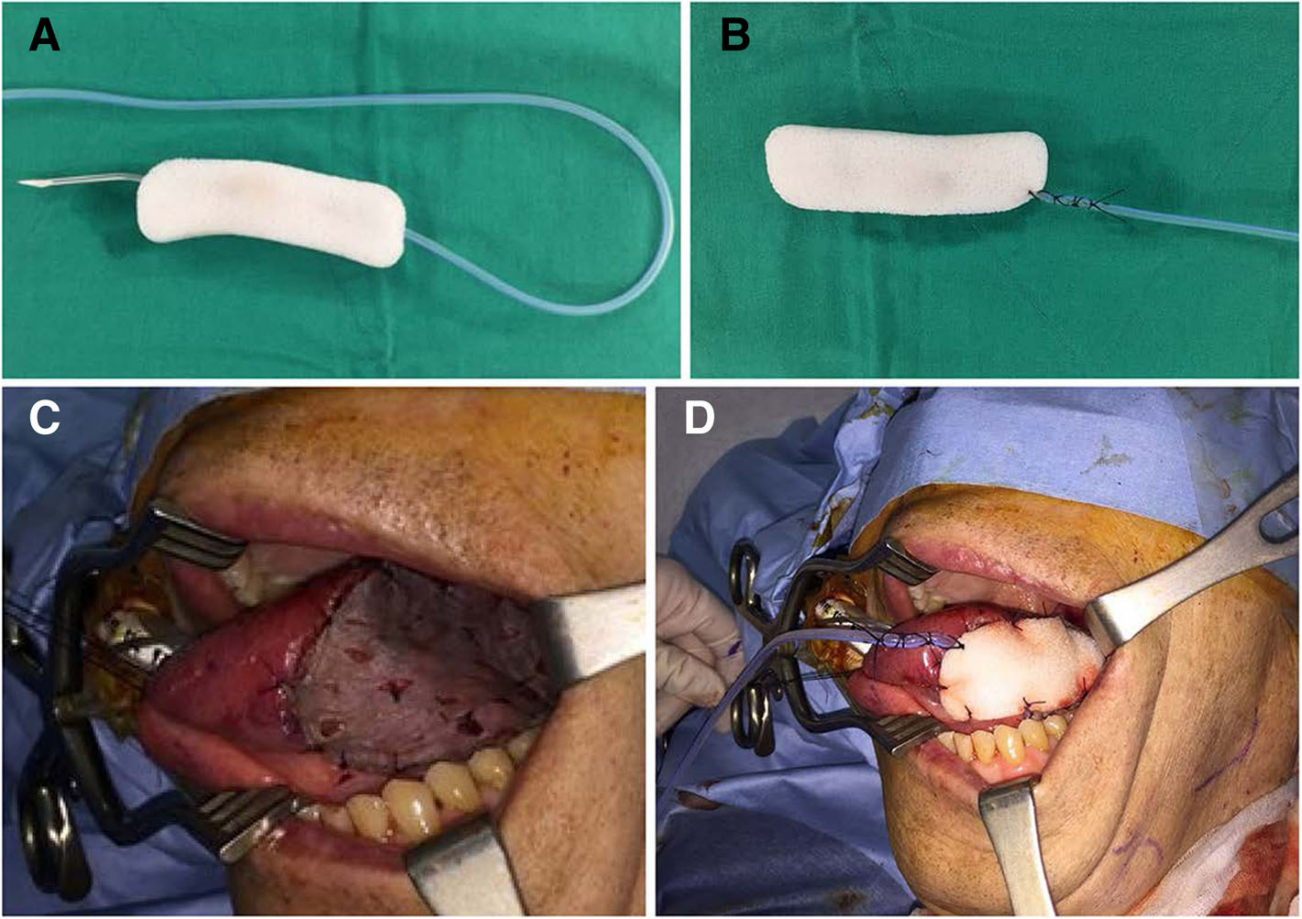
A procedure of bolster dressing with continuous suction over the skin graft in an oral cavity wound. **a** A polyvinyl alcohol sponge (Merocel®) was inserted with a trocar of a 100 cc Jackson-Pratt (JP) drain. Several suction holes in the line were placed within the sponge. **b** The drain was anchored with a tagging suture. **c** The skin graft was placed over the intraoral defect with a pie-crust technique. **d** JP-tagged Merocel® was placed on the Gelfoam® and anchored with tie-over sutures

After the same procedure of applying a skin graft with the pie-crusted technique in the conventional bolster method, a sterile, absorbable gelatin sponge USP (Gelfoam®, Pharmacia & Upjohn Co., MI, USA) was put on the skin graft. A Merocel® was designed to fit the skin graft size and a 100 cc Jackson-Pratt (JP) drain (CardinalHealth, OH, USA) was inserted inside the Merocel® sponge with tagging sutures (Fig. 1a-b). Then, the JP-tagged sponge was placed on the gelatin sponge-covered skin graft and tie-over suturing was performed to compress the graft (Fig. 1c-d).

The suction line of the JP apparatus was directly connected to the suction device, without using the collection bulb. Although sometimes we used the wall suction device (when the patient was in his bed), the main suction device, exerting the negative pressure on the sponge, was a portable suction device (Curasys®, Atozbio Co, Korea). This was the vacuum device for the negative pressure dressing (a vacuum assisted closure therapy system).

**Fig. 2:**
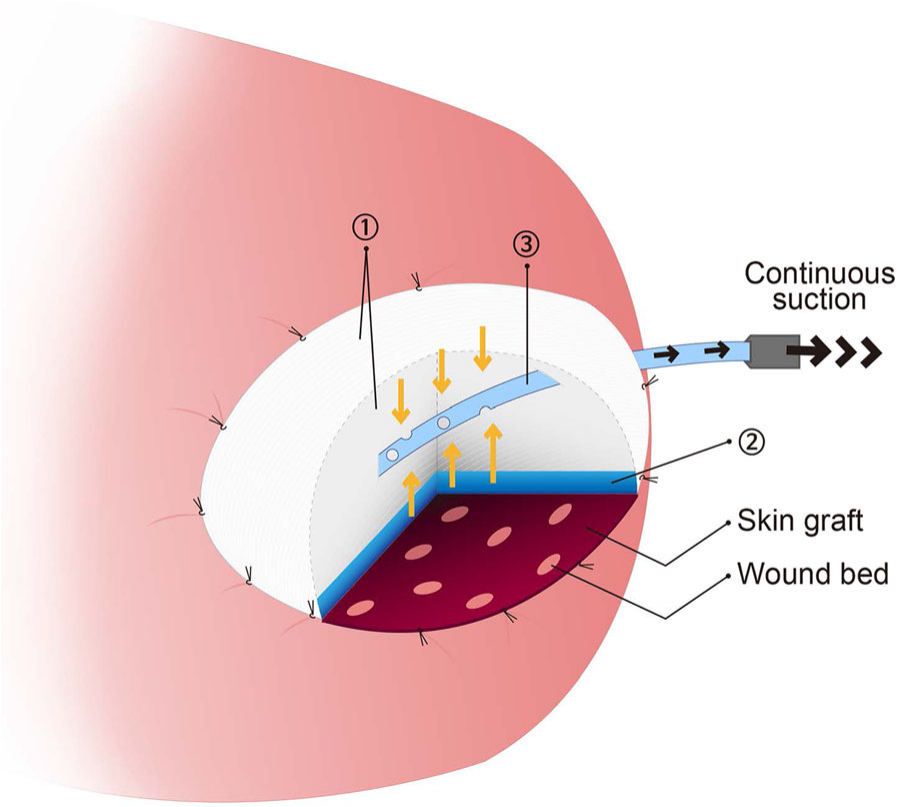
Schematic drawing of a bolster dressing with continuous suction over an intraoral skin graft

We adjusted the JP drain pressure in the range of 100 to 180 mmHg to effectively remove the accumulated saliva in each patient (Fig. 2). Daily assessment of the skin graft was performed to determine the proper time of removal of the bolster dressing.

### Evaluation of successful engraftment

**Fig. 3:**
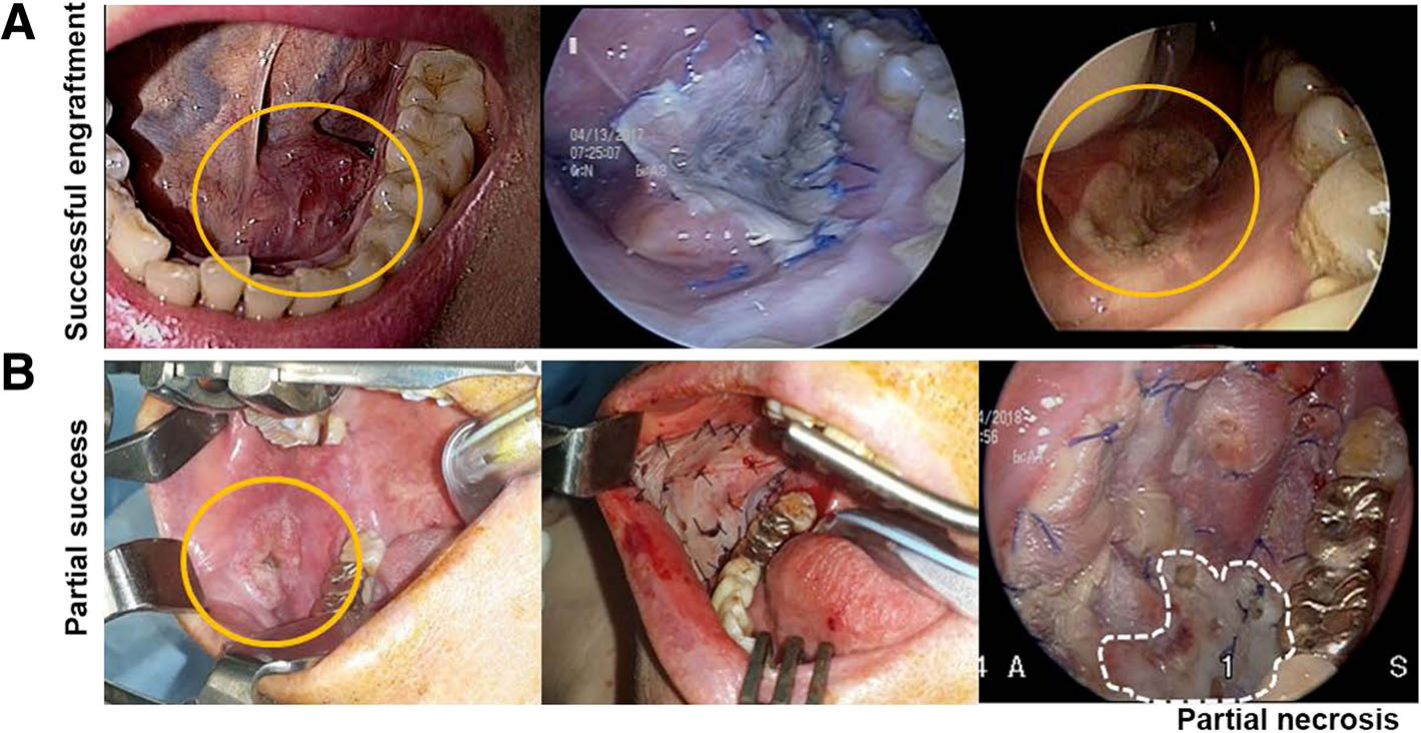
Representative findings of successful and partially successful engraftment in the modified bolster group with serial follow-ups (pre-operative status, immediate after removing bolster, post operative 1–2 months). **a** Successful engraftment in modified bolster group. **b** Partial successful engraftment in modified bolster group

Graft status was evaluated based on graft healing and size by visual inspection at the time of wound healing (at postoperative 1-4 months). In this study, we regarded it as successful engraftment if there was no definite dehiscence or detachment of the skin graft from the wound bed. When there was necrotic debris or exudates at a portion of the graft site which consequently led to an incomplete healing of the skin graft, we regarded it as a partial engraftment. If there was no remnant skin graft in the bed, we classified it as a graft failure. Representative cases of successful engraftment and partial success are shown in Fig. 3.

## Results

### Patient demographics and clinical information

A total of 31 patients with a median age of 62 years (range 29–79) who were reconstructed with skin graft after resection of oral cavity cancer were reviewed in this study. Of these, 21 patients used the conventional bolster method and 10 patients used our modified bolster method. There was no statistical difference between two groups in age, sex, site of primary cancer and graft type (Additional file 1: Table S2). In terms of clinical stage, there was a statistically significant difference between the two groups (*p* = 0.045). Nine out of 21 patients in the conventional bolster group and 9 out of 10 patients in the modified bolster group had clinical T1 tumors. Eleven out of 21 patients in the conventional bolster group and 1 out of 10 in the modified bolster group had clinical T2 tumors. However, the area (size) of skin graft was not different between the two groups (*p* = 0.31). All pathological diagnoses were squamous cell carcinoma except one (intermediate grade mucoepidermoid carcinoma). Individual clinical characteristics of all patients in two groups are listed in Table 1 and Additional file 1: Table S1.

**Table 1:** Case series and outcomes (Modified bolster with continuous suction)

Case No.	Gender/Age	Primary site	Pathology	pT	Size of defect (cm)	Type of skin graft	Duration of continuous suction (day)	Outcomes
1	F/ 57	FOM	MEC	pT2	3.5 × 2	FTSG	6	S
2	M/ 49	Tongue	SCC	pT1	5 × 3.5	FTSG	4	PS
3	M/ 53	FOM	SCC	pT1	7 × 3^a^	FTSG	3	F
4	M/ 62	FOM	SCC	pT1	4 × 2	FTSG	4	S
5	M/ 53	FOM	SCC	pT1	4.5 × 2.5	STSG	6	S
6	M/ 43	Tongue	SCC	pT1	3.5 × 3	FTSG	10	S
7	M/ 79	Tongue	SCC	pT1	7 × 4^a^	STSG	7	S
8	M/ 59	FOM	SCC	pT1	5 × 4	STSG	7	S
9	M/ 56	FOM	SCC	pT1	5 × 4	STSG	4	S
10	M/ 74	Buccal	SCC	pT1	4.5 × 3.5	STSG	4	PS

*FOM* Floor of the mouth, *MEC* Mucoepidermoid carcinoma (intermediate grade), *SCC* Squamous cell carcinoma, *FTSG* Full thickness skin graft, *STSG* Split thickness skin graft (thickness 12/1000 in), *S* Successful engraftment of skin graft, *PS* Partially successful engraftment of skin graft and partial necrosis (less than 1/3), *F* Failure and necrosis of skin graft

^a^Wide resection including tumor and mucosal dysplasia area

### Success rate and size of the skin graft

**Fig. 4:**
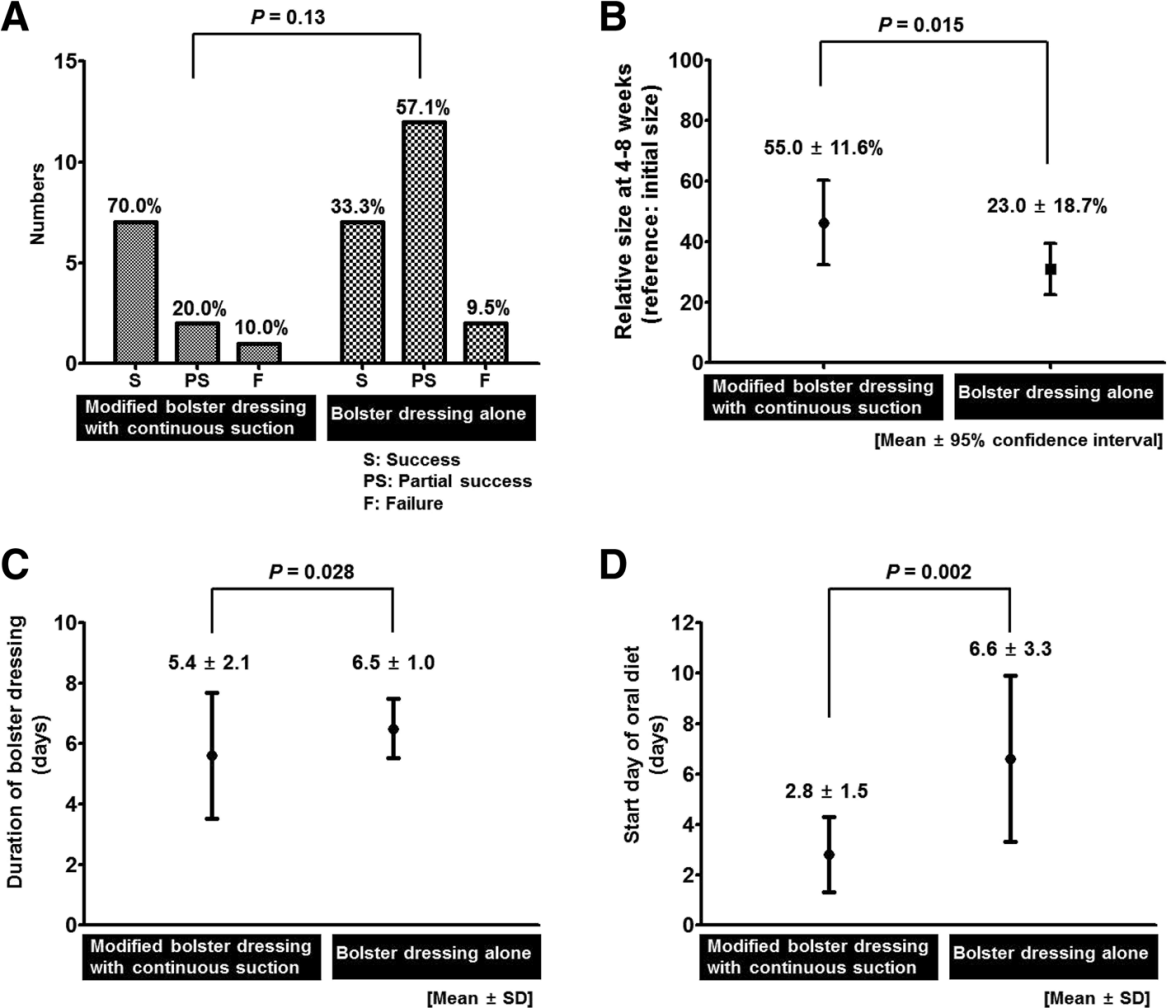
Comparison of outcomes between the modified bolster group and the control group. **a** Success rate of skin graft, **b** Relative size of the skin graft at the time of healing, **c** Duration of bolster dressing, **d** Start day of oral diet

The overall success rate of oral skin graft in the group with bolster dressing and continuous suction was not different from that of the control group (bolster dressing alone) (90.0 and 90.5%). However, partial necrosis (partial success) in the engrafted skin was observed frequently in the control group (57.1% versus 20.0% in the modified bolster group) (Fig. 4a). As a result, the relative engrafted area (reference: the initial area of skin graft) was significantly higher in the modified bolster group (55.0 ± 11.6% versus 23.0 ± 18.7%, *p* = 0.015) (Fig. 4b). These findings suggest that a bolster dressing with continuous suction could enhance skin engraftment in the whole area, and prevent skin detachment in the wound margin.

### The duration of bolster dressing and start of oral diet

The average time (days from the operation day) of bolster (Merocel®) placement was significantly shorter in the modified bolster group (5.6 days) than in the conventional bolster group (6.5 days) (*p* = 0.028) (Fig. 4c). Although the ideal time of removing a bolster dressing has not been strictly described, 5 days have been considered as the average time point [13].

In the modified bolster group, patients started an oral diet (soft fluid diet) earlier than in the conventional bolster group (2.8 ± 1.5 days and 6.6 ± 3.3 days, *p* = 0.002) (Fig. 4d). While patients in the conventional bolster group tended to start an oral diet after bolster removal, patients in the modified bolster group started an oral diet with the bolster still in place. Thus, modified bolster dressing can help patients to start an early oral diet.

### Graft failures in the modified bolster group

In the modified bolster group, successful engraftments were achieved in all patients except three (No. 2, No. 3 and No.10 in Table 1). For Case No.2 and No.10, the skin graft was partially detached (1/3), while the remaining one was successfully engrafted. For Case No.3, the patient had well adapted skin graft after removal of Merocel®. However, he experienced a bleeding event from the surgical wound, and his skin graft was detached from the bed. The skin graft eventually developed necrosis.

## Discussion

Oral skin graft is one of the most effective reconstruction methods when the defect is superficial and an epithelial covering is desired to restore a barrier and prevent tethering of oral mucosa. Successful skin grafting could help patients achieve excellent articulation and swallowing function after healing [14]. The most attractive point of skin graft reconstruction is that it is an easy and quick method compared to other reconstructions and does not require multiple resources. Success rates of skin graft (split thickness) in the oral cavity, tongue, and floor of the mouth have been reported to be 71, 77 and 87%, respectively [15].

The most common reason for graft failure is incomplete hemostasis at the recipient site [16]. Similarly, in our study, most of the partially successful engraftments had exudates and necrotic debris around the skin graft, which caused the skin graft to detach from its bed. In the oral cavity, saliva retained under the skin graft could be another risk factor. To overcome this, making multiple small incisions in the graft, the so-called ‘pie crust incision’ has been suggested [17].

Recently, a negative pressure dressing (for example, vacuum-assisted closure therapy) has been introduced to facilitate skin graft adherence [5, 18–20]. However, it is hard to apply a negative pressure dressing in the oral cavity because it has limited space and an irregular recipient bed. Instead, a conventional bolster dressing (tie-over suture) is generally used to secure an intraoral skin graft. Although a conventional bolster dressing decreases sheering forces within the graft, it cannot prevent blood or saliva accumulation under the graft. Thus, we designed a modified method to remove saliva or exudates under the skin graft by applying continuous suction to the conventional method.

Our modification is simple and effective in removing saliva accumulation under skin graft. Two or three suction holes in the JP drain are buried in the Merocel®. Therefore, saliva or secretions around the oral cavity wound can be sucked out through the drain. In our study, we applied a continuous suction pressure ranging from 100 to180 mmHg. Though this pressure seems to be quite strong, it does not produce direct pressure on the oral cavity wound because the sponge (Merocel®) with a suction drain was placed in an open space (not negative pressure). Also, when we apply suction pressure via a JP drain, the sponge collapses around the holes in the drain and reduces the actual suction pressure on the wound and the skin graft. If saliva was sticky, we elevated the drain pressure up to 180 mmHg. Thus, the important role of suction drain is to effectively remove the absorbed secretions in the sponge, drying it to absorb other secretions. Furthermore, when starting an oral diet with continuous suction, no patient complained about the high suction pressure.

We experienced malfunctions of continuous suction in some patients due to obstruction of the drain. The main cause of drain obstruction was local collapse of the sponge around small holes in the JP drain. In such cases, repositioning the drain line or lowering the power used for the continuous suction was needed.

The duration of the continuous suction with Merocel® was decided based on the status of skin graft adherence by regular examination of skin graft through the gap between the Merocel® sponge and the wound bed. If there was no definite dehiscence or detachment in skin graft, we removed Merocel® and continuous suction. When removing the dressing, the soft gelatin layer (Gelfoam®) could play a role as a good physical buffer to prevent the skin graft from accidental detachment. With our method, we could reduce the average duration of bolster to 5 days from the operation day.

In our experience, applying continuous suction was harder for tongue lesions than for floor of the mouth lesions. This might be because the tongue is more mobile. On the floor of the mouth, saliva accumulation is more prominent than at a tongue lesion. Considering this, our technique could be more effective particularly in the floor of the mouth area. Moreover, in patients with oral cavity cancer, there would be a higher chance of saliva accumulation because of decreased swallowing function (before or after surgery). This will likely have a negative influence on successful engraftment.

In summary, the modified bolster technique can help the skin graft adapt to the intra oral wound bed by removing saliva and exudates. Removing exudates prevents skin graft detachment from the wound bed, which can result in necrosis of the skin graft. In terms of patient convenience, no patient had complained about maintaining the oral suction line during the post-operative period.

As we mentioned before, the modified bolster technique can advance the starting time of an oral diet, which can eventually reduce the duration of hospital stay. Furthermore, allowing the patient to try an oral diet earlier can increase patient comfort.

One of the limitations of this study is the small number of patients of each group, especially in the modified bolster group, so that it is difficult to generalize the results. Another limitation is that this study has a selection bias because of the retrospective nature of the data collection. We expect a further prospective study with a large number of patients to be performed to support our preliminary results.

## Conclusions

This study suggests the technical feasibility of using our modified method to remove exudate and saliva under an intraoral skin graft. Further trials are needed to verify our method. Furthermore, detailed technical refinement is also needed to control saliva or exudates more effectively. Though this study has some limitations, these results suggest a new technical modification to the conventional method. This might trigger future focus on the effect of saliva control on the success of intra oral skin graft.

## Additional file

Additional file 1: **Table S1.** Clinical characteristics of patients with the conventional bolster dressing (controls). **Table S2.** Comparison of baseline variables between the modified bolster dressing group and the control group. (DOCX 25 kb)
