# Supplementary material for: Modified bolster dressing with continuous suction improves skin graft survival for an oral cavity wound
Source: J Otolaryngol Head Neck Surg. 2018 Nov 14;47:68. doi: 10.1186/s40463-018-0314-7 (PMC6234621; doi:10.1186/s40463-018-0314-7)
Supplement: Supplementary file 1 — Table S1. Clinical characteristics of patients with the conventional bolster dressing (controls). Table S2. Comparison of baseline variables between the modified bolster dressing group and the control group. (DOCX 25 kb) [file 40463_2018_314_MOESM1_ESM.docx]

**Table S1.** Clinical characteristics of patients with the conventional bolster dressing (controls)

| Case No. | Gender/Age | Primary site | Pathology | pT | Size of defect  (cm) | Type of skin graft | Duration of bolster  (day) | Outcomes |
| --- | --- | --- | --- | --- | --- | --- | --- | --- |
| 1 | M/67 | FOM | SCC | pT2 | 5×3 | STSG | 8 | PS |
| 2 | M/68 | Tongue | SCC | pT1 | 4×2 | FTSG | 8 | PS |
| 3 | F/70 | Tongue | SCC | pT2 | 7×3 | STSG | 7 | PS |
| 4 | F/66 | Tongue | SCC | pT1 | 4.5×3 | STSG | 7 | S |
| 5 | M/45 | Tongue | SCC | pT1 | 4.5×2 | STSG | 7 | S |
| 6 | M/55 | FOM | SCC | pT1 | 8×4 | FTSG | 7 | PS |
| 7 | M/62 | Tongue | SCC | pT3 | 8×5 | STSG | 7 | S |
| 8 | M/66 | FOM | SCC | pT1 | 5×3 | STSG | 8 | PS |
| 9 | M/41 | Tongue | SCC | pT2 | 10×4 | STSG | 5 | PS |
| 10 | M/70 | FOM | SCC | pT2 | 5×3 | STSG | 6 | PS |
| 11 | M/62 | FOM | SCC | pT2 | 5×4 | FTSG | 6 | S |
| 12 | M/66 | FOM | SCC | pT2 | 7×5 | STSG | 5 | PS |
| 13 | F/64 | Tongue | SCC | pT2 | 6×3.5 | STSG | 7 | S |
| 14 | M/79 | Tongue | SCC | pT2 | 10×5 | STSG | 6 | PS |
| 15 | M/41 | Tongue | SCC | pT2 | 5.5×4 | STSG | 5 | S |
| 16 | M/57 | Tongue | SCC | pT1 | 3×2 | STSG | 6 | PS |
| 17 | F/62 | Tongue | SCC | pT2 | 3×3 | STSG | 5 | PS |
| 18 | M/29 | Tongue | SCC | pT2 | 7×5 | STSG | 7 | PS |
| 19 | M/37 | Tongue | SCC | pT1 | 5×5 | STSG | 7 | S |
| 20 | F/35 | Tongue | SCC | pT1 | 4.5×3 | FTSG | 6 | F |
| 21 | M/62 | Tongue | SCC | pT1 | 4×2.5 | FTSG | 7 | F |

FOM: Floor of mouth
MEC: Mucoepidermoid carcinoma (intermediate grade)
SCC: Squamous cell carcinoma

FTSG: Full thickness skin graft

STSG: Split thickness skin graft (thickness 12/1000 inches)

S: Successful engraftment of skin graft

PS: Partially successful engraftment of skin graft and partial necrosis (less than ⅓)

F: Failure and necrosis of skin graft

**Table S2.** Comparison of baseline variables between the modified bolster dressing group and the control group.

| Baseline characteristics | Modified bolster with continuous suction group  (n = 10) | Conventional bolster dressing alone  (Control group)  (n = 21) | *P*-value |
| --- | --- | --- | --- |
| Gender (M:F) (No. %) | 9:1 (90.0:10.0) | 16:5 (76.2:23.8) | 0.35 |
| Age (Mean, SD, years) | 56.8 ± 10.0 | 57.3 ± 13.7 | 0.914 |
| Primary sites (No.) |  |  | 0.06 |
| Tongue | 3 | 15 |  |
| Floor of the mouth | 6 | 6 |  |
| Buccal | 1 | 0 |  |
| Types of skin graft (No.) |  |  | 0.14 |
| Split thickness | 5 | 16 |  |
| Full thickness | 5 | 5 |  |
| pT classification |  |  | 0.045 |
| T1:T2:T3 | 9:1:0 | 9:11:1 |  |
| Skin grafted area (Mean, SD, cm^2^) | 15.9 ± 6.7 | 22.7 ± 12.5 | 0.31 |

[END]
